# Supplementary material for: Managing university e-learning environments and academic achievement in the United Arab Emirates: An instructor and student perspective
Source: PLoS One. 2022 May 12;17(5):e0268338. doi: 10.1371/journal.pone.0268338 (PMC9097996; doi:10.1371/journal.pone.0268338)
Supplement: S1 Appendix — (DOCX) [file pone.0268338.s001.docx]

**Appendix 1**

| VARIABLES |  | QUESTION |
| --- | --- | --- |
| Academic Engagement | AE1 | I used to Find ways to make the course interesting to me |
|  | AE2 | I'm thinking about the course between class meetings |
|  | AE3 | I really desiring to learn the material |
|  | AE4 | I'm having fun in class |
| Digital Readiness | DR1 | I can use keywords to search information in the internet for my course work. |
|  | DR2 | I can interact with classmates using real-time communication tools (e.g., video conferencing tools or messengers). |
|  | DR1 | I can share my files with classmates using online software. |
|  | DR2 | I can collaborate with classmates using online software. |
| E-learning Adoption | EA1 | I have the knowledge necessary to use the digital learning system. |
|  | EA2 | I have control over the digital learning system |
|  | EA3 | I have the resources necessary to use the digital learning system. |
| E-learning Environment_ | EE1 | The Technology is in place to communicate effectively with the students and conduct the classes remotely. |
|  | EE2 | The students are aware to use the e-learning and distance learning technology |
| E-learning Attitude | ET1 | I feel positively about digital learning. |
|  | ET2 | Studying with digital learning is a good idea. |
|  | ET3 | All things considered, using the digital learning system on campus is beneficial to me. |
| Instructor Attitude | IT1 | The instructor presented the material well and clearly. |
|  | IT2 | The instructor started and ended the lectures on time and was regular |
|  | IT3 | The instructor encouraged interaction with students listened to them and responded to their questions. |
| Academic Achievement | GPA |  |
