# Supplementary material for: Managing university e-learning environments and academic achievement in the United Arab Emirates: An instructor and student perspective
Source: PLoS One. 2022 May 12;17(5):e0268338. doi: 10.1371/journal.pone.0268338 (PMC9097996; doi:10.1371/journal.pone.0268338)
Supplement: Attachment — Submitted filename: PONE-D-21-40699-Review.docx [file pone.0268338.s002.docx]

**Introduction**

*The research background needed to be explained more clearly. What practical problem was the research aimed at solving? (Page 8, Lines 50 - 52).

* In this study, how is E-learning related to traditional learning? This sentence “The present research question focuses on how to preserve students’ traditional in-person academic achievements and educational experiences……” is too abrupt. (Page 8, Lines 59 - 61).

** Literature review**

The literature review summarized research on online learning or E-learning and elaborated the contribution of this study. However, the influencing factors mentioned in the study were not explained clearly. Specifically, despite stating the influencing factors, the study did not explain the relationship between factors and academic achievement. In the Research model and hypotheses section, the study mentioned “The model is based on the literature review findings for the following factors……”, but the arguments were not justified enough in the part of Literature review.

** Research model and hypotheses**

*Analyzing previous literature and making research hypotheses will help to clarify the relationships between variables.

* Research hypotheses should be developed first, then a model should be developed to explain the relationship between the variables analyzed. The hypotheses should also be labeled on Figure 1.

**Methodology and Data Analysis**

*A descriptive statistical analysis is best presented as a table.

* An analysis of model fit is missing from this study.

** Discussion and implications**

The discussion section is too weak. The arguments needed to be focused on results and elaborated with more studies/research.
