# Supplementary material for: Managing university e-learning environments and academic achievement in the United Arab Emirates: An instructor and student perspective
Source: PLoS One. 2022 May 12;17(5):e0268338. doi: 10.1371/journal.pone.0268338 (PMC9097996; doi:10.1371/journal.pone.0268338)
Supplement: Attachment — Submitted filename: Final rebutal letter.docx [file pone.0268338.s006.docx]

**Answers to academic editor comments and improvements.**

I appreciate the extensive revision efforts taken by the authors. As a result, the quality of the paper has been significantly increased, and I don't see any major issues regarding the paper content.

Thank you very much for all the suggestions and recommendations to enhance the present research. All of them will clearly enhance the quality of the present research.

However, before I can accept this paper, the authors need to further refine the format of the manuscript to meet Plos One requirements. The guidelines and sample paper of Plos One are attached in this e-mail for your reference.

The format of the manuscript has been adapted to meet Plos One requirements, based on the guidelines and sample paper.

Also, The color and style of proposed model in Figure 1 and validated model in Figure 2 are drastically different. I would suggest make them consistent in design and style.

The color and style of proposed model in Figure 1 and validated model in Figure 2 have been redesigned to present a consistent style.

Thank you again for all the support and guidance along the entire process. We look forward to provide value to the journal with the present research.

Very kind regards,
